# Factors related to age at natural menopause in China: results from the China Kadoorie Biobank

**DOI:** 10.1097/GME.0000000000001829

**Published:** 2021-08-02

**Authors:** Meng Wang, Christiana Kartsonaki, Yu Guo, Jun Lv, Wei Gan, Zheng-Ming Chen, Li-Ming Li, Chong-Gao Hu, Ling Yang, Min Yu

**Affiliations:** 1 Department of Non-Communicable Disease Control and Prevention, Zhejiang Provincial Center for Disease Control and Prevention, Hangzhou, China; 2 Clinical Trial Service Unit and Epidemiological Studies Unit (CTSU), Nuffield Department of Population Health, University of Oxford, Oxford, UK; 3 Chinese Academy of Medical Sciences, Dong Cheng District, Beijing, China; 4 Department of Epidemiology, School of Public Health, Peking University Health Science Center, Haidian District, Beijing, China; 5 School of Biotechnology and Health Sciences, Wuyi University, Jiangmen, China.

**Keywords:** Age at menopause, Chinese, Risk factors, Women

## Abstract

**Objectives:**

The aim of this study was to investigate the potentially modifiable factors affecting age at natural menopause (ANM) in Chinese women.

**Methods:**

We used cross-sectional data from the China Kadoorie Biobank study which that recruited 0.5 million (0.3 million women) Chinese adults aged 30 to 79 from 2004 to 2008. Multinomial logistic regression models were used to examine the relationships between ANM and various factors recorded at baseline.

**Results:**

Among 87,349 postmenopausal women, the mean ANM (SD) was 48.7 (4.3) years. Older age, being a housewife, earlier menarche, and passive smoking were associated with both premature menopause (PM, ie, ANM <40 years) and early menopause (EM, ie, ANM between 40 and 44 years). A higher odds for EM was observed in women who were widowed (odds ratio: 1.10, 95% confidence interval: 1.04-1.16), had spontaneous abortions (1.33 [1.05-1.69]), current regular smoking (1.19 [1.07-1.37]), and frequent spicy food intake (1.11 [1.05-1.08]). Higher socioeconomic status; later first birth; more live births and induced abortions; longer breastfeeding; tea drinking, as well as intakes of meat, fruits, dairy, and soybean products; and increased body mass index gain were inversely associated with PM and/or EM. In contrast, women who had more pregnancies, occasional alcohol drinking, higher levels of physical activity or body mass index, vitamin intake, and hypertension were more likely to have a later age at menopause (LM, ie, ANM ≥53 years).

**Conclusions:**

This large epidemiological study found a wide range of sociodemographic, lifestyle, dietary, and reproductive factors related to PM, EM, and LM in Chinese women.

Menopause is an important event in a woman's reproductive history and the age at natural menopause (ANM) has held great public health interest due to its implications for numerous health outcomes. Several studies found that premature/early menopause (EM) is associated with higher risk of type 2 diabetes,^1^ cardiovascular disease,^2^ all-cause mortality,^3^ worse cognitive function,^4^ and osteoporosis and fracture.^5,6^ In contrast, late menopause is associated with higher risk of cancers in the breast^7^ and endometrium.^8^ Therefore, identifying the related factors to the ANM may shed light on the etiology, early monitoring, and prevention of these relevant diseases in later life.

Menopausal age varies greatly between and within populations.^9^ Previous research has shown that menopause is triggered by a low threshold number of predetermined follicles in the ovary^10^; however, factors that can modify the rate of follicle decline and thus affect the onset of menopause are not fully understood. Except for the genetic contributions to the variation in ANM,^11,12^ there are potential effects of other reproductive and lifestyle factors, such as parity and smoking.^13^

However, most existing evidence came from Western studies, and little is known in China, where women's lifestyle and reproductive characteristics are significantly different from those in Western women. Based on the nationwide China Kadoorie Biobank (CKB) study, we aimed to examine the relationships between ANM and a wide range of sociodemographic, lifestyle, dietary, and reproductive factors in Chinese women.

## METHODS

### Study design and population

Details regarding the CKB study design and population have been published elsewhere.^14^ Briefly, the baseline survey was conducted from 2004 to 2008 and recruited 512,715 Chinese adults (302,522 women) aged 30 to 79 from 10 diverse regions in China. Data on participants’ sociodemographic characteristics, dietary and lifestyle behaviors, medical history, and for women only, the history of reproductive characteristics, relevant surgery treatment, and oral contraceptive (OC) use were collected using an interviewer-administered laptop-based questionnaire. Physical measurements including various anthropometry measurements (eg, bodyweight, standing height, waist circumference, and blood pressure) were undertaken by trained health workers.

Self-reported ANM was the outcome of interest. On the baseline questionnaire, women were asked whether they had their menopause with the following response options: (1) no; (2) yes, currently; (3) yes, had menopause. Women who had menopause were then asked the age of completion of menopause (open response). In this study, women without menstruation for 12 months or longer were defined as postmenopausal. In categorical analysis, ANM was grouped as age less than 40 (“premature menopause”, PM), 40 to 44 (EM), 45 to 47, 48 to 50 (reference category), 51 to 52, and 53 years or older (“later age at menopause”, LM).^15^

Exposure factors considered in the cross-sectional study included: (i) sociodemographic characteristics included age, area, marriage status, education, occupation, and household income; (ii) lifestyle behavior factors included tea and alcohol drinking, active and passive smoking, physical activity, and the duration of pesticide storage at home; (iii) dietary intake variables included frequency of consuming coarse cereals, meat, poultry, seafood, fresh eggs, soybean and dairy products, preserved vegetables, fresh fruits and vegetables, spicy food, vitamins and minerals, and experienced severe food shortage over 1 year preceding the survey; (iv) physical measurements included body mass index (BMI), BMI change per year from age 25 years, waist circumference, and blood pressure; and (v) reproductive characteristics included age at menarche and first birth, parous status, OC use, number of pregnancies, live births, spontaneous and induced abortions, and breastfeeding duration for each live birth. The details of these exposure factors could be found in the supplementary baseline questionnaire (Supplemental Digital Content).

### Statistical analysis

Among 302,522 women recruited in the CKB study, 146,160 women were excluded because they were premenopausal (*n* = 128,721) or perimenopausal (*n* = 14,828), had missing data on menopause (*n* = 47), or had a history of surgical menopause (*n* = 1,240) or cancer (*n* = 1,324) at baseline. To avoid any potential distortion of the distribution of ANM among the younger age group which included pre-, peri-, and postmenopausal women, the main analysis was confined to women aged 57 years or older of whom 99% reported being postmenopausal (ie, 67,865 women <57 years old at baseline were further excluded). The extreme top and bottom 0.5% of menopausal age values were also excluded (*n* = 1,148). After these exclusions, 87,349 postmenopausal women remained in the main analysis.

The age-adjusted mean ANM for each category of each exposure variable was calculated. Multivariable linear regression models were used to estimate the adjusted mean differences and their 95% confidence intervals (CIs) for ANM between different categories of each exposure variable. To further estimate adjusted odds ratios (ORs) and 95% CIs for PM, EM, and LM according to each category of exposure variables, we used the multinomial (polytomous) logistic regression models. Based on prior knowledge, the statistical models were adjusted for age (57-60, 61-64, 65-69, ≥70 years), area (rural, urban), education (no formal school, primary school, middle school, high school, college/university), household income (<10k, 10-20k [10,000-19,999], 20-35k [20,000-34,999], ≥ 35k yuan), smoking (never, occasional, current regular), BMI (underweight, normal weight, overweight, obesity),^16^ age at menarche (≤12, 13-14, 15-16, 17-18, ≥19 years) and number of live births (1, 2, 3, ≥4). Tests for linear trend were conducted by modeling each exposure as a continuous variable. The Bonferroni correction was used to adjust for multiple comparisons. Finally, to evaluate the robustness of our estimates, we conducted the sensitivity analysis with different categories of ANM: <40 (PM), 40-44 (EM), 45-52 (reference category), and ≥53 years (LM). All analyses were performed using SAS version 9.4 (SAS Institute, Inc., Cary, NC) and R version 4.0.2 (The R Foundation for Statistical Computing). All statistical tests were based on the two-sided 5% level of significance.

## RESULTS

Among 87,349 postmenopausal women, the mean (SD) age at baseline was 64.5 (5.2) years. The range of reported ANM was 32.0 to 58.0 years, with a mean of 48.7 (4.3) years, and a median of 49.0 years. Approximately 3.2% of women had PM, 10.9% EM, and 16.7% LM. 49.8% of the postmenopausal women were from urban areas and 40.9% had no formal education. Few women were current regular smokers (4.7%) or alcohol drinkers (3.4%) and 8.5% women had ever used OC. Very few women were nulliparous (1.2%) or, among parous women, never breastfed their children (1.7%) during their reproductive period. Compared with women with a later ANM, women with earlier menopause were, on average, slightly older and leaner at baseline, more likely to be less educated, smoked more, and had a higher proportion of nulliparity, less use of OC, younger age at first birth, and lack of breastfeeding (Table 1).

**TABLE 1:** Baseline characteristics of China Kadoorie Biobank women according to age at natural menopause

		Age at natural menopause (y)					
Characteristics	Overall	<40	40-44	45-47	48-50	51-52	≥53
No. of women	87,349	2,794	9,514	16,660	30,581	13,225	14,575
Mean age at baseline (y)	64.5	66.2	65.4	64.8	64.6	64.0	63.6
Birth cohorts (%)							
1920s-1930s	34.7	48.3	41.4	37.0	35.6	30.1	27.1
1940s	62.6	49.8	56.6	60.3	61.8	66.9	69.6
1950s	2.7	1.9	2.0	2.7	2.6	3.0	3.3
Urban resident (%)	49.8	47.5	40.8	49.2	50.4	51.2	54.0
No formal school (%)	40.9	52.2	46.7	41.6	40.5	40.0	36.0
Lifestyle factors and physical measurements, % or mean							
Current regular smoker	4.7	6.3	6.2	5.3	4.7	4.0	3.6
Current regular drinker	3.4	3.9	3.9	3.6	3.2	3.4	3.1
BMI (kg/m^2^)	23.9	23.5	23.5	23.8	23.8	24.2	24.5
Overweight (24.0-27.9 kg/m^2^)	33.8	31.4	31.3	33.0	32.9	35.3	37.2
Obesity (≥28.0 kg/m^2^)	13.9	11.5	11.9	13.4	13.2	15.1	16.6
Waist circumference (cm)	81.1	80.0	80.3	80.8	80.9	81.5	82.3
Physical activity, MET (h/d)	14.5	13.9	14.3	14.5	14.3	15.1	14.7
Reproductive factors, % or mean							
Age at menarche (y)	16.1	16.1	16.0	16.1	16.2	16.1	16.2
Nulliparous	1.2	2.9	1.7	1.5	1.2	0.9	0.8
Oral contraceptive pill used	8.5	5.48	6.37	8.22	8.44	9.48	9.89
No. of live births^*a*^	3.4	3.7	3.7	3.5	3.4	3.3	3.3
Age at first birth (y)^*a*^	22.4	21.9	22.0	22.3	22.4	22.4	22.7
Never breastfed^*a*^	1.7	2.4	2.1	1.8	1.7	1.5	1.7
Breastfeeding per child (mo)^*a*^	14.8	15.0	15.0	14.9	14.5	14.8	14.8

BMI, body mass index; MET, metabolic equivalents of task.

a Among parous women only.

**FIG. 1:**
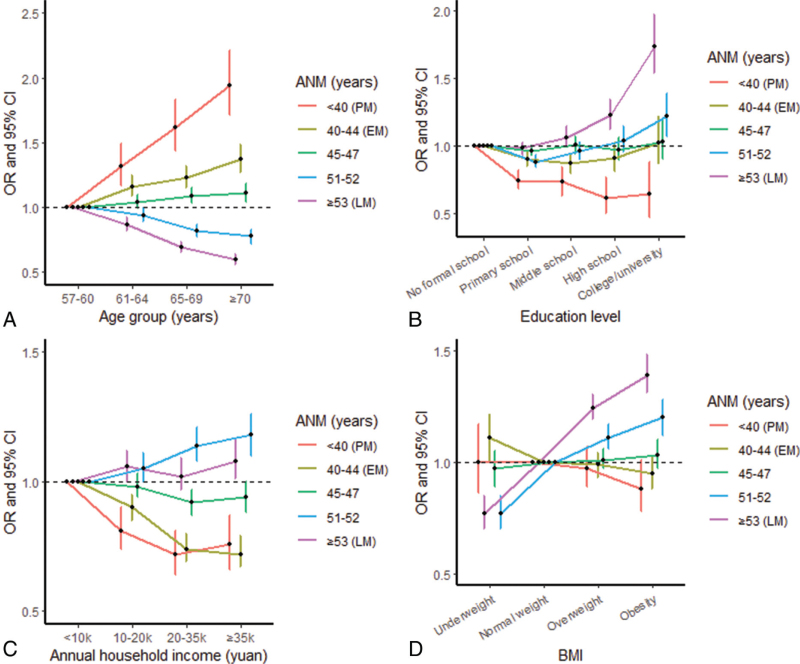
Associations of ANM with factors of age, education level, household income, and BMI. Specifically, **(A)** with age; **(B)** with education level; **(C)** with household income; **(D)** with BMI. Dots represent the ORs compared with the reference group of menopausal age (48-50 years). Vertical lines indicate the corresponding 95% CIs. ANM, age at natural menopause; BMI, body mass index; CI, confidence interval; EM, early menopause (ANM between 40 and 44 years); LM, later age at menopause (ANM ≥53 years); OR, odds ratio; PM, premature menopause (ANM < 40 years).

**FIG. 2:**
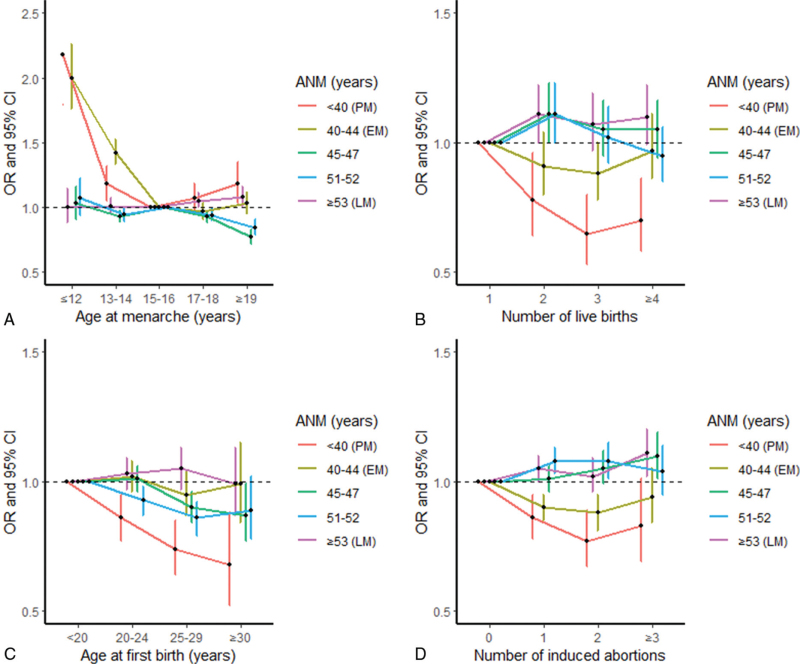
Associations of ANM with age at menarche, number of live births, age at first birth, and number of induced abortions. Specifically, **(A)** with age at menarche; **(B)** with number of live births; **(C)** with age at first birth; **(D)** with number of induced abortions. Dots represent the OR compared with the reference group of menopausal age (48-50 years). Vertical lines indicate the corresponding 95% CIs. ANM, age at natural menopause; BMI, body mass index; CI, confidence interval; EM, early menopause (ANM between 40 and 44 years); LM, later age at menopause (ANM ≥53 years); OR, odds ratio; PM, premature menopause (ANM <40 years).

Specifically, compared with the reference category of ANM (48-50 years), higher odds were present in women who were housewives (vs unemployed/retired/other), with OR of 1.18 (95% CI: 1.04-1.34) for PM and 1.23 (1.14-1.34) for EM; in older age groups (vs 57-60 years) for both PM (with OR between 1.32-1.94, *P*_trend_ < 0.001) and EM (1.16-1.37, *P*_trend_ < 0.001); with earlier menarche (≤12 and 13-14 vs 15-16 years): 1.18-2.18 for PM and 1.42-2.00 for EM; experienced passive smoking (occasional and current regular vs never): 1.16-1.20 (*P*_trend_ = 0.006) for PM and 1.13-1.16 (*P*_trend_ < 0.001) for EM, respectively. A higher odds for EM was also observed in women who were widowed (vs married, OR = 1.10 [1.04-1.16]), had three or more spontaneous abortions (vs none, 1.33 [1.05-1.69]), current regular smoking (vs never, 1.19 [1.07-1.37]), and frequent spicy food intake (6-7 days/week vs never, 1.11 [1.05-1.08]) (Tables 2-5, Fig. 1A and Fig. 2A).

**TABLE 2:** Adjusted mean age at natural menopause and associations with sociodemographic characteristics of women

Characteristics	No. of women (%)	Age at menopause, (y), mean (SD)^*a*^	Mean difference ß (95% CI)	<40 y OR (95% CI)	40-44 y OR (95% CI)	45-47 y OR (95% CI)	51-52 y OR (95% CI)	≥53 y OR (95% CI)
Age (y)								
57-60	28,372 (32.5)	49.2 (4.1)	0.00	1.00	1.00	1.00	1.00	1.00
61-64	20,898 (23.9)	48.8 (4.3)	−0.33 (−0.41 to −0.26)	1.32 (1.17-1.49)	1.16 (1.09-1.25)	1.04 (0.99-1.10)	0.94 (0.89-0.99)	0.87 (0.82-0.92)
65-69	21,912 (25.1)	48.3 (4.3)	−0.75 (−0.83 to −0.67)	1.62 (1.44-1.83)	1.23 (1.15-1.32)	1.09 (1.03-1.15)	0.82 (0.77-0.87)	0.69 (0.65-0.73)
≥70	16,167 (18.5)	47.9 (4.4)	−1.07 (−1.16 to −0.98)	1.94 (1.71-2.21)	1.37 (1.27-1.48)	1.11 (1.04-1.18)	0.78 (0.72-0.83)	0.60 (0.56-0.64)
*P*_trend_			<0.001	<0.001	<0.001	<0.001	<0.001	<0.001
Area								
Rural	43,882 (50.2)	48.3 (4.4)	0.00	1.00	1.00	1.00	1.00	1.00
Urban	43,467 (49.8)	49.0 (4.2)	0.30 (0.23-0.36)	0.95 (0.87-1.03)	0.72 (0.68-0.76)	0.94 (0.90-0.98)	0.94 (0.89-0.98)	1.06 (1.01-1.11)
*P*_trend_			0.001	0.218	<0.001	0.005	0.006	0.022
Marriage status								
Married	66,011 (75.6)	48.7 (4.2)	0.00	1.00	1.00	1.00	1.00	1.00
Widowed	20,797 (23.8)	48.5 (4.4)	−0.12 (−0.19 to −0.05)	0.97 (0.88-1.06)	1.10 (1.04-1.16)	1.02 (0.97-1.07)	0.96 (0.91-1.01)	0.97 (0.93-1.03)
Separated/divorced/unmarried	541 (0.6)	48.8 (4.5)	0.11 (−0.26 to 0.49)	1.40 (0.86-2.30)	1.21 (0.88-1.67)	1.00 (0.76-1.31)	1.07 (0.81-1.42)	1.34 (1.04-1.73)
*P*_trend_			0.002	0.483	<0.001	0.242	0.122	0.703
Highest education								
No formal school	35,706 (40.9)	48.4 (4.4)	0.00	1.00	1.00	1.00	1.00	1.00
Primary school	32,298 (37.0)	48.6 (4.2)	0.15 (0.08 to 0.21)	0.74 (0.68-0.82)	0.90 (0.85-0.95)	0.96 (0.92-1.01)	0.88 (0.84-0.93)	0.98 (0.93-1.02)
Middle school	11,155 (12.7)	48.9 (4.1)	0.27 (0.17 to 0.37)	0.73 (0.63-0.84)	0.87 (0.79-0.94)	1.01 (0.94-1.07)	0.96 (0.89-1.03)	1.06 (0.99-1.14)
High school	5,766 (6.6)	49.2 (4.0)	0.47 (0.34 to 0.60)	0.61 (0.50-0.76)	0.91 (0.81-1.02)	0.97 (0.89-1.06)	1.04 (0.95-1.14)	1.23 (1.12-1.34)
College/university	2,424 (2.8)	49.6 (4.0)	0.80 (0.61 to 0.99)	0.64 (0.47-0.88)	1.02 (0.86-1.22)	1.03 (0.90-1.18)	1.22 (1.07-1.39)	1.74 (1.54-1.97)
*P*_trend_			<0.001	<0.001	0.003	0.314	0.112	<0.001
Occupation								
Unemployed/retired/other	31,899 (36.5)	49.1 (4.1)	0.00	1.00	1.00	1.00	1.00	1.00
Manager/technologist/business	1,764 (2.0)	48.9 (4.2)	0.01 (−0.19 to 0.22)	1.32 (0.98-1.79)	1.13 (0.93-1.36)	1.05 (0.91-1.21)	1.17 (1.02-1.36)	1.10 (0.95-1.26)
Housewives	25,343 (29.0)	48.5 (4.4)	−0.14 (−0.24 to −0.05)	1.18 (1.04-1.34)	1.23 (1.14-1.34)	0.98 (0.92-1.04)	0.98 (0.91-1.05)	1.07 (1.00-1.14)
Agriculture related/factory worker	28,343 (32.5)	48.3 (4.3)	−0.09 (−0.20 to 0.02)	1.21 (1.04-1.40)	1.24 (1.13-1.36)	1.07 (1.00-1.16)	1.10 (1.02-1.19)	1.12 (1.04-1.21)
*P*_trend_			0.022	0.004	<0.001	0.037	0.014	0.001
Annual household income (yuan)								
<10k	31,816 (36.5)	48.3 (4.5)	0.00	1.00	1.00	1.00	1.00	1.00
10-20k	24,572 (28.1)	48.7 (4.2)	0.29 (0.21-0.36)	0.81 (0.74-0.90)	0.90 (0.85-0.95)	0.98 (0.94-1.03)	1.05 (1.00-1.11)	1.06 (1.00-1.12)
20-35k	18,361 (21.0)	49.0 (4.1)	0.49 (0.41-0.57)	0.72 (0.64-0.81)	0.74 (0.69-0.80)	0.92 (0.87-0.97)	1.14 (1.08-1.21)	1.02 (0.97-1.09)
≥35k	12,600 (14.4)	49.1 (4.1)	0.54 (0.45-0.64)	0.76 (0.66-0.87)	0.72 (0.67-0.79)	0.94 (0.88-1.00)	1.18 (1.10-1.26)	1.08 (1.01-1.16)
*P*_trend_			<0.001	<0.001	<0.001	0.002	<0.001	0.021

ß and OR were adjusted for age, area, education, annual household income, smoking, body mass index (BMI), age at menarche, and number of live births, except for the same variable. When calculating the OR, menopausal age of 48 to 50 years was used as the reference group. *P* < 0.001 did not change after Bonferroni corrections.

CI, confidence interval; OR, odds ratio.

a Adjusted for age at baseline (continuous), except for the age variable.

**TABLE 3:** Adjusted mean age at natural menopause and associations with reproductive characteristics of women

Characteristics	No. of women (%)	Age at menopause (y), mean (SD)^*a*^	Mean difference ß (95% CI)	<40 y OR (95% CI)	40-44 y OR (95% CI)	45-47 y OR (95% CI)	51-52 y OR (95% CI)	≥53 y OR (95% CI)
Age at menarche (y)								
≤12	2,563 (2.9)	47.9 (4.7)	−0.86 (−1.03 to −0.69)	2.18 (1.79-2.66)	2.00 (1.76-2.26)	1.03 (0.91-1.16)	1.07 (0.94-1.22)	1.00 (0.88-1.14)
13-14	15,607 (17.9)	48.5 (4.3)	−0.28 (−0.36 to −0.19)	1.18 (1.05-1.32)	1.42 (1.33-1.52)	0.93 (0.88-0.98)	0.95 (0.89-1.00)	1.01 (0.95-1.07)
15-16	31,506 (36.1)	48.7 (4.2)	0.00	1.00	1.00	1.00	1.00	1.00
17-18	28,390 (32.5)	48.7 (4.2)	0.09 (0.03-0.16)	1.07 (0.97-1.18)	0.97 (0.91-1.03)	0.93 (0.88-0.97)	0.94 (0.90-0.99)	1.05 (1.00-1.11)
≥19	9,283 (10.6)	48.7 (4.4)	0.10 (0.001-0.20)	1.18 (1.03-1.35)	1.03 (0.95-1.12)	0.77 (0.72-0.83)	0.84 (0.79-0.91)	1.08 (1.01-1.16)
*P*_trend_			<0.001	0.002	<0.001	<0.001	<0.001	0.004
Parous status								
Nulliparous	1,077 (1.2)	47.3 (4.8)	0.00	1.00	1.00	1.00	1.00	1.00
Parous	86,272 (98.8)	48.7 (4.3)	1.37 (1.11-1.62)	0.40 (0.31-0.51)	0.69 (0.58-0.84)	0.79 (0.67-0.94)	1.25 (1.02-1.55)	1.38 (1.12-1.70)
*P*_trend_			<0.001	<0.001	<0.001	0.003	0.018	0.002
Oral contraceptives use								
Never	79,944 (91.5)	48.6 (4.3)	0.00	1.00	1.00	1.00	1.00	1.00
Ever	7,405 (8.5)	49.1 (4.0)	0.24 (0.13-0.34)	0.78 (0.66-0.92)	0.88 (0.80-0.96)	0.99 (0.93-1.07)	1.05 (0.98-1.13)	1.07 (0.99-1.14)
*P*_trend_			<0.001	0.004	0.006	0.871	0.191	0.071
Number of pregnancies^*b*^								
1	1,593 (1.9)	48.3 (4.5)	0.00	1.00	1.00	1.00	1.00	1.00
2	8,058 (9.3)	48.6 (4.2)	0.23 (−0.05 to 0.52)	0.94 (0.65-1.36)	1.13 (0.89-1.44)	1.04 (0.85-1.26)	1.25 (1.02-1.54)	1.24 (1.02-1.52)
3	18,377 (21.2)	48.8 (4.1)	0.51 (0.22-0.79)	0.72 (0.49-1.06)	1.06 (0.83-1.36)	1.03 (0.84-1.25)	1.40 (1.14-1.73)	1.33 (1.09-1.63)
≥4	58,497 (67.6)	48.6 (4.3)	0.56 (0.27-0.85)	0.67 (0.45-0.98)	1.02 (0.79-1.30)	1.03 (0.84-1.25)	1.36 (1.11-1.68)	1.34 (1.10-1.64)
*P*_trend_			<0.001	<0.001	0.040	0.636	0.018	0.009
Number of live births^*c*^								
1	3,755 (4.4)	48.7 (4.3)	0.00	1.00	1.00	1.00	1.00	1.00
2	20,352 (23.6)	48.9 (4.1)	0.25 (0.11-0.40)	0.78 (0.64-0.96)	0.91 (0.80-1.04)	1.11 (1.00-1.23)	1.11 (1.00-1.23)	1.11 (1.00-1.22)
3	26,605 (30.8)	48.8 (4.1)	0.30 (0.15−0.44)	0.65 (0.53-0.80)	0.88 (0.78-1.00)	1.05 (0.95-1.16)	1.02 (0.92-1.14)	1.07 (0.97-1.19)
≥4	35,560 (41.2)	48.4 (4.4)	0.17 (0.02-0.32)	0.70 (0.58-0.86)	0.97 (0.86-1.11)	1.05 (0.94-1.16)	0.95 (0.85-1.06)	1.10 (0.99-1.22)
*P*_trend_			0.438	0.002	0.063	0.132	<0.001	0.218
Age at first birth (y)^*c*^								
<20	17,027 (19.8)	48.4 (4.4)	0.00	1.00	1.00	1.00	1.00	1.00
20-24	48,518 (56.2)	48.6 (4.3)	0.06 (−0.02 to 0.14)	0.86 (0.77-0.95)	1.02 (0.96-1.08)	1.01 (0.96-1.06)	0.93 (0.87-0.98)	1.03 (0.97-1.09)
25-29	17,725 (20.5)	49.0 (4.1)	0.19 (0.08-0.29)	0.74 (0.64-0.85)	0.95 (0.87-1.04)	0.90 (0.84-0.96)	0.86 (0.79-0.92)	1.05 (0.97-1.13)
≥30	3,002 (3.5)	49.0 (4.1)	0.19 (0.01-0.38)	0.68 (0.52-0.88)	0.99 (0.84-1.15)	0.87 (0.77-0.99)	0.89 (0.78-1.02)	0.99 (0.87-1.13)
*P*_trend_			<0.001	<0.001	0.139	<0.001	<0.001	0.183
Number of spontaneous abortions^*b*^								
0	75,884 (87.7)	48.7 (4.3)	0.00	1.00	1.00	1.00	1.00	1.00
1	8,163 (9.4)	48.8 (4.3)	0.19 (0.10-0.29)	0.80 (0.70-0.93)	0.97 (0.90-1.05)	0.95 (0.89-1.01)	0.98 (0.92-1.06)	1.08 (1.01-1.16)
2	1,798 (2.1)	48.5 (4.4)	0.04 (−0.16 to 0.24)	1.02 (0.79-1.33)	1.08 (0.92-1.26)	1.00 (0.87-1.14)	1.04 (0.90-1.21)	1.12 (0.97-1.29)
≥3	680 (0.8)	48.1 (4.4)	−0.37 (−0.69 to −0.05)	0.96 (0.62-1.49)	1.33 (1.05-1.69)	1.17 (0.95-1.45)	1.13 (0.88-1.44)	0.99 (0.77-1.27)
*P*_trend_			0.072	0.032	0.058	0.480	0.259	0.010
Number of induced abortions^*b*^								
0	48,410 (56.0)	48.5 (4.4)	0.00	1.00	1.00	1.00	1.00	1.00
1	21,910 (25.3)	48.9 (4.1)	0.22 (0.15-0.29)	0.86 (0.78-0.95)	0.90 (0.85-0.95)	1.01 (0.96-1.06)	1.08 (1.03-1.13)	1.05 (1.00-1.10)
2	10,990 (12.7)	49.0 (4.1)	0.22 (0.13-0.31)	0.77 (0.67-0.89)	0.88 (0.81-0.95)	1.05 (0.99-1.12)	1.08 (1.01-1.15)	1.02 (0.96-1.09)
≥3	5,215 (6.0)	48.9 (4.1)	0.17 (0.05-0.29)	0.83 (0.69-1.01)	0.94 (0.84-1.05)	1.10 (1.01-1.19)	1.04 (0.95-1.14)	1.11 (1.02-1.20)
*P*_trend_			<0.001	<0.001	<0.001	0.007	0.009	0.010
Duration of breastfeeding per child (mo)^*c*^								
Never breastfed	1,504 (1.7)	48.4 (4.5)	0.00	1.00	1.00	1.00	1.00	1.00
1-6	4,987 (5.8)	48.5 (4.4)	0.37 (0.13-0.61)	0.76 (0.56-1.03)	0.78 (0.65-0.95)	0.94 (0.80-1.11)	1.14 (0.94-1.37)	1.01 (0.85-1.21)
7-12	41,763 (48.4)	48.8 (4.2)	0.63 (0.41-0.84)	0.61 (0.46-0.80)	0.69 (0.58-0.83)	0.85 (0.73-0.98)	1.11 (0.94-1.31)	1.02 (0.87-1.20)
13-18	18,917 (21.9)	48.7 (4.3)	0.66 (0.44-0.88)	0.65 (0.49-0.87)	0.74 (0.62-0.89)	0.94 (0.80-1.09)	1.23 (1.04-1.46)	1.15 (0.97-1.35)
19-24	12,721 (14.8)	48.5 (4.4)	0.51 (0.28-0.74)	0.77 (0.58-1.02)	0.77 (0.64-0.93)	0.97 (0.83-1.13)	1.21 (1.01-1.44)	1.08 (0.91-1.27)
≥25	6,380 (7.4)	48.3 (4.4)	0.43 (0.19-0.67)	0.80 (0.59-1.08)	0.88 (0.73-1.06)	1.07 (0.91-1.26)	1.26 (1.05-1.51)	1.17 (0.98-1.39)
*P*_trend_			0.172	0.008	0.004	<0.001	<0.001	<0.001

ß and OR were adjusted for age, area, education, annual household income, smoking, body mass index (BMI), age at menarche, and number of live births, except for the same variable. When calculating the OR, menopausal age of 48 to 50 years was used as the reference group. *P* < 0.001 did not change after Bonferroni corrections.

CI, confidence interval; OR: odds ratio.

a Adjusted for age at baseline (continuous), except for the age variable.

b Among ever pregnant women.

c Among parous women only.

**TABLE 4:** Adjusted mean age at natural menopause and associations with lifestyle characteristics of women

Characteristics	No. of women (%)	Age at menopause (y), mean (SD)^*a*^	Mean difference ß (95% CI)	<40 y OR (95% CI)	40-44 y OR (95% CI)	45-47 y OR (95% CI)	51-52 y OR (95% CI)	≥53 y OR (95% CI)
Tea drinking								
Never	46,793 (53.6)	48.6 (4.3)	0.00	1.00	1.00	1.00	1.00	1.00
Occasional	20,311 (23.2)	48.7 (4.2)	0.07 (0.004-0.15)	0.84 (0.76-0.93)	0.99 (0.93-1.05)	0.98 (0.93-1.02)	0.93 (0.89-0.98)	1.04 (0.99-1.09)
Current regular	20,245 (23.2)	48.7 (4.3)	0.11 (0.04-0.18)	0.84 (0.76-0.93)	0.98 (0.92-1.04)	0.86 (0.82-0.90)	0.88 (0.84-0.93)	1.03 (0.98-1.08)
*P*_trend_			0.001	<0.001	0.261	<0.001	<0.001	0.117
Alcohol intake								
Never	62,226 (71.2)	48.6 (4.3)	0.00	1.00	1.00	1.00	1.00	1.00
Occasional	22,164 (25.4)	48.8 (4.2)	0.10 (0.03-0.16)	1.04 (0.94-1.14)	0.99 (0.93-1.04)	1.08 (1.03-1.13)	1.04 (0.99-1.09)	1.11 (1.06-1.16)
Current regular	2,959 (3.4)	48.4 (4.4)	−0.06 (−0.22 to 0.10)	1.17 (0.95-1.45)	1.09 (0.96-1.24)	1.09 (0.98-1.22)	1.08 (0.96-1.21)	1.02 (0.90-1.14)
*P*_trend_			0.054	0.083	0.418	0.001	0.031	0.001
Smoking								
Never	79,275 (90.8)	48.7 (4.26)	0.00	1.00	1.00	1.00	1.00	1.00
Occasional	3,956 (4.5)	48.5 (4.38)	−0.07 (−0.20 to 0.07)	1.00 (0.83-1.21)	1.09 (0.98-1.22)	1.13 (1.04-1.24)	1.15 (1.04-1.27)	1.02 (0.92-1.13)
Current regular	4,118 (4.7)	48.1 (4.39)	−0.40 (−0.53 to −0.26)	1.19 (1.00-1.40)	1.19 (1.07-1.31)	1.13 (1.03-1.23)	0.95 (0.86-1.05)	0.88 (0.79-0.97)
*P*_trend_			<0.001	0.034	<0.001	0.001	0.542	0.019
Passive smoking								
Never	23,854 (27.3)	48.9 (4.16)	0.00	1.00	1.00	1.00	1.00	1.00
Occasional	21,626 (24.8)	48.6 (4.31)	−0.13 (−0.21 to −0.05)	1.20 (1.08-1.35)	1.13 (1.06-1.21)	1.05 (1.00-1.11)	1.02 (0.96-1.08)	1.03 (0.98-1.09)
Current regular	41,869 (47.9)	48.5 (4.31)	−0.20 (−0.27 to −0.13)	1.16 (1.05-1.28)	1.16 (1.09-1.23)	1.05 (1.00-1.10)	0.94 (0.90-0.99)	1.00 (0.96-1.06)
*P*_trend_			<0.001	0.006	<0.001	0.047	0.007	0.786
Physical activity								
Low	26,901 (30.8)	48.6 (4.3)	0.00	1.00	1.00	1.00	1.00	1.00
Middle	38,609 (44.2)	48.7 (4.2)	0.05 (−0.02 to 0.16)	0.95 (0.86-1.04)	1.01 (0.95-1.07)	1.05 (1.00-1.10)	1.04 (0.99-1.09)	1.06 (1.01-1.11)
High	21,839 (25.0)	48.5 (4.3)	0.07 (−0.01 to 0.15)	1.00 (0.89-1.11)	0.99 (0.93-1.06)	1.08 (1.02-1.14)	1.12 (1.06-1.19)	1.07 (1.01-1.13)
*P*_trend_			0.035	0.511	0.576	0.004	<0.001	0.009
Duration of pesticide storage at home (mo)								
0	60,241 (69.0)	48.8 (4.3)	0.00	1.00	1.00	1.00	1.00	1.00
1-6	3,127 (3.6)	48.0 (4.4)	−0.21 (−0.37 to −0.05)	0.98 (0.79-1.22)	1.03 (0.91-1.16)	1.05 (0.95-1.17)	0.94 (0.83-1.06)	0.90 (0.80-1.01)
7-12	23,981 (27.4)	48.4 (4.3)	0.05 (−0.03 to 0.12)	1.06 (0.96-1.18)	0.96 (0.90-1.02)	1.05 (0.99-1.10)	1.16 (1.09-1.22)	0.98 (0.92-1.03)
*P*_trend_			0.146	0.137	0.085	0.048	<0.001	0.229

ß and OR were adjusted for age, area, education, annual household income, smoking, body mass index (BMI), age at menarche, and number of live births, except for the same variable. When calculating the OR, menopausal age of 48 to 50 years was used as the reference group. *P* < 0.001 did not change after Bonferroni corrections.

CI, confidence interval; OR, odds ratio.

a Adjusted for age at baseline (continuous), except for the age variable.

**TABLE 5:** Adjusted mean age at natural menopause and associations with dietary characteristics of women

Characteristics	No. of women (%)	Age at menopause (y), mean (SD)^*a*^	Mean difference ß (95% CI)	<40 y OR (95% CI)	40-44 y OR (95% CI)	45-47 y OR (95% CI)	51-52 y OR (95%CI)	≥53 y OR (95%CI)
Coarse cereals								
Never	22,193 (25.4)	48.5 (4.3)	0.00	1.00	1.00	1.00	1.00	1.00
<1 d/wk	39,730 (45.5)	48.7 (4.3)	−0.02 (−0.10 to 0.05)	0.96 (0.87-1.06)	1.01 (0.95-1.07)	0.95 (0.91-1.00)	0.97 (0.92-1.03)	0.97 (0.92-1.02)
1-3 d/wk	13,868 (15.9)	49.0 (4.2)	0.01 (−0.09 to 0.11)	0.94 (0.82-1.08)	1.04 (0.96-1.13)	0.96 (0.90-1.03)	0.97 (0.90-1.04)	1.04 (0.98-1.12)
≥4 d/wk	11,558 (13.2)	48.5 (4.3)	−0.08 (−0.18 to 0.02)	1.35 (1.18-1.54)	1.17 (1.08-1.27)	1.20 (1.13-1.29)	1.12 (1.04-1.21)	1.14 (1.06-1.23)
*P*_trend_			0.089	<0.001	<0.001	<0.001	0.006	<0.001
Meat								
Never	5,537 (6.3)	48.1 (4.6)	0.00	1.00	1.00	1.00	1.00	1.00
<1 d/wk	12,602 (14.4)	48.3 (4.4)	0.12 (−0.01 to 0.26)	0.83 (0.70-0.98)	0.93 (0.84-1.03)	0.94 (0.86-1.03)	1.08 (0.97-1.20)	0.90 (0.81-0.99)
1-3 d/wk	34,050 (39.0)	48.6 (4.3)	0.29 (0.17-0.41)	0.72 (0.62-0.84)	0.85 (0.78-0.94)	0.89 (0.82-0.96)	1.03 (0.93-1.13)	0.96 (0.88-1.05)
≥4 d/wk	35,160 (40.3)	48.9 (4.2)	0.37 (0.24-0.49)	0.63 (0.54-0.74)	0.81 (0.74-0.90)	0.80 (0.74-0.87)	0.95 (0.87-1.05)	0.94 (0.85-1.03)
*P*_trend_			<0.001	<0.001	<0.001	<0.001	0.001	0.307
Poultry								
Never	34,940 (40.0)	48.3 (4.4)	0.00	1.00	1.00	1.00	1.00	1.00
<1 d/wk	31,899 (36.5)	48.8 (4.2)	0.23 (0.16-0.30)	0.87 (0.79-0.95)	0.88 (0.83-0.93)	0.92 (0.88-0.97)	1.07 (1.02-1.13)	1.00 (0.96-1.05)
1-3 d/wk	19,821 (22.7)	49.0 (4.1)	0.23 (0.14-0.31)	0.83 (0.73-0.93)	0.82 (0.76-0.88)	0.91 (0.86-0.97)	0.93 (0.88-0.99)	0.98 (0.93-1.04)
≥4 d/wk	689 (0.8)	49.0 (4.0)	0.27 (−0.05 to 0.60)	0.57 (0.32-1.00)	0.71 (0.53-0.95)	0.87 (0.70-1.08)	0.81 (0.64-1.02)	0.86 (0.69-1.07)
*P*_trend_			<0.001	<0.001	<0.001	<0.001	0.010	0.145
Seafood								
Never	27,448 (31.4)	48.2 (4.5)	0.00	1.00	1.00	1.00	1.00	1.00
<1 d/wk	20,636 (23.6)	48.7 (4.2)	0.33 (0.25-0.42)	0.80 (0.72-0.90)	0.78 (0.73-0.84)	0.88 (0.83-0.93)	1.07 (1.01-1.14)	0.94 (0.89-1.00)
1-3 d/wk	32,321 (37.0)	48.9 (4.2)	0.39 (0.30-0.47)	0.77 (0.68-0.87)	0.78 (0.73-0.84)	0.82 (0.77-0.87)	1.01 (0.94-1.07)	0.99 (0.93-1.05)
≥4 d/wk	6,944 (8.0)	49.0 (4.2)	0.37 (0.24-0.50)	0.77 (0.64-0.92)	0.74 (0.66-0.82)	0.69 (0.63-0.75)	0.89 (0.81-0.98)	0.91 (0.83-1.00)
*P*_trend_			<0.001	<0.001	<0.001	<0.001	0.015	0.108
Fresh eggs								
Never	10,488 (12.0)	48.3 (4.5)	0.00	1.00	1.00	1.00	1.00	1.00
<1 d/wk	18,629 (21.3)	48.5 (4.3)	0.14 (0.04-0.24)	0.83 (0.73-0.95)	0.94 (0.87-1.02)	0.97 (0.90-1.04)	1.07 (0.99-1.16)	0.95 (0.88-1.02)
1-3 d/wk	37,805 (43.3)	48.7 (4.2)	0.17 (0.08-0.26)	0.88 (0.78-0.99)	0.90 (0.83-0.97)	1.02 (0.96-1.08)	1.09 (1.01-1.17)	1.00 (0.93-1.07)
≥4 d/wk	20,427 (23.4)	48.9 (4.2)	0.23 (0.13-0.33)	0.80 (0.70-0.92)	0.91 (0.84-0.99)	0.99 (0.93-1.07)	1.08 (1.00-1.17)	1.04 (0.97-1.12)
*P*_trend_			<0.001	0.006	0.006	0.360	0.036	0.019
Fresh vegetables								
≤3 d/wk	1,490 (1.7)	48.3 (4.3)	0.00	1.00	1.00	1.00	1.00	1.00
4-6 d/wk	3,321 (3.8)	48.5 (4.3)	0.08 (−0.18 to 0.34)	0.93 (0.64-1.37)	1.03 (0.84-1.25)	1.04 (0.87-1.24)	1.10 (0.90-1.35)	1.03 (0.85-1.25)
Daily	82,538 (94.5)	48.7 (4.3)	0.01 (−0.21 to 0.23)	1.49 (1.09-2.04)	1.06 (0.90-1.25)	1.15 (1.00-1.34)	1.24 (1.05-1.47)	1.14 (0.97-1.35)
*P*_trend_			0.384	<0.001	0.248	0.004	0.001	0.009
Soybean products								
Never	10,208 (11.7)	48.4 (4.4)	0.00	1.00	1.00	1.00	1.00	1.00
<1 d/wk	24,742 (28.3)	48.4 (4.4)	0.02 (−0.08 to 0.11)	0.95 (0.83-1.08)	0.95 (0.88-1.03)	0.96 (0.90-1.03)	1.01 (0.94-1.09)	0.93 (0.87-1.00)
1-3 d/wk	43,371 (49.7)	48.8 (4.2)	0.13 (0.04-0.22)	0.95 (0.83-1.08)	0.87 (0.81-0.94)	0.96 (0.90-1.03)	1.08 (1.00-1.16)	0.95 (0.89-1.02)
≥4 d/wk	9,028 (10.3)	49.0 (4.1)	0.28 (0.16-0.41)	0.97 (0.82-1.16)	0.80 (0.72-0.89)	0.93 (0.86-1.02)	1.11 (1.01-1.21)	1.02 (0.94-1.11)
*P*_trend_			<0.001	0.327	<0.001	0.061	0.001	0.238
Preserved vegetables								
Never	17,847 (20.5)	48.7 (4.4)	0.00	1.00	1.00	1.00	1.00	1.00
<1 d/wk	25,885 (29.6)	48.7 (4.2)	0.05 (−0.03 to 0.13)	0.85 (0.75-0.95)	0.91 (0.85-0.97)	0.96 (0.91-1.02)	1.00 (0.94-1.06)	0.91 (0.86-0.97)
1-3 d/wk	22,637 (25.9)	48.6 (4.3)	0.03 (−0.05 to 0.11)	0.79 (0.70-0.89)	0.91 (0.85-0.98)	0.96 (0.91-1.02)	0.98 (0.92-1.04)	0.90 (0.85-0.96)
≥4 d/wk	20,980 (24.0)	48.7 (4.3)	−0.06 (−0.15 to 0.02)	0.98 (0.88-1.11)	0.98 (0.91-1.06)	1.07 (1.00-1.13)	1.11 (1.04-1.19)	0.93 (0.87-0.98)
*P*_trend_			0.034	0.258	0.352	0.012	0.001	0.005
Fresh fruits								
Never	6,726 (7.7)	48.2 (4.5)	0.00	1.00	1.00	1.00	1.00	1.00
<1 d/wk	30,493 (34.9)	48.4 (4.4)	0.11 (0.001-0.23)	0.95 (0.83-1.10)	0.95 (0.87-1.04)	0.99 (0.92-1.07)	1.07 (0.98-1.17)	1.02 (0.94-1.11)
1-3 d/wk	25,009 (28.6)	48.7 (4.2)	0.24 (0.13-0.36)	0.77 (0.66-0.90)	0.87 (0.80-0.96)	0.99 (0.91-1.07)	1.12 (1.02-1.22)	1.01 (0.93-1.11)
≥4 d/wk	25,121 (28.8)	49.0 (4.1)	0.31 (0.19-0.43)	0.80 (0.68-0.94)	0.90 (0.81-0.99)	1.02 (0.94-1.11)	1.11 (1.01-1.21)	1.15 (1.05-1.25)
*P*_trend_			<0.001	<0.001	0.002	0.197	0.016	<0.001
Dairy products								
Never	57,208 (65.5)	48.5 (4.3)	0.00	1.00	1.00	1.00	1.00	1.00
<1 d/wk	9,605 (11.0)	48.7 (4.2)	0.03 (−0.07 to 0.12)	0.89 (0.77-1.01)	0.98 (0.91-1.06)	0.99 (0.93-1.05)	0.92 (0.86-0.98)	1.00 (0.94-1.07)
1-3 d/wk	6,996 (8.0)	49.0 (4.1)	0.18 (0.06-0.29)	0.77 (0.65-0.91)	0.86 (0.78-0.95)	0.92 (0.85-0.99)	0.93 (0.86-1.01)	0.99 (0.91-1.07)
≥4 d/wk	13,540 (15.5)	49.2 (4.1)	0.24 (0.15- 0.33)	0.79 (0.69-0.91)	0.90 (0.83-0.97)	0.97 (0.92-1.03)	0.98 (0.92-1.05)	1.10 (1.04-1.17)
*P*_trend_			<0.001	<0.001	<0.001	0.058	0.120	0.003
Vitamins intake								
No	82,774 (94.8)	48.6 (4.3)	0.00	1.00	1.00	1.00	1.00	1.00
Yes	4,575 (5.2)	49.2 (4.1)	0.32 (0.19- 0.45)	0.90 (0.73-1.10)	0.91 (0.81-1.02)	1.00 (0.92-1.09)	1.19 (1.08-1.30)	1.17 (1.07-1.27)
*P*_trend_			<0.001	0.310	0.096	0.985	<0.001	0.001
Minerals intake								
No	77,155 (88.3)	48.6 (4.3)	0.00	1.00	1.00	1.00	1.00	1.00
Yes	10,194 (11.7)	48.9 (4.2)	−0.01 (−0.10 to 0.08)	0.97 (0.85-1.11)	1.05 (0.97-1.13)	1.05 (0.99-1.12)	1.03 (0.96-1.10)	1.04 (0.98-1.11)
*P*_trend_			0.781	0.644	0.215	0.109	0.420	0.213
Experienced severe food shortage								
No	54,471 (62.4)	48.7 (4.2)	0.00	1.00	1.00	1.00	1.00	1.00
Yes	32,878 (37.6)	48.6 (4.3)	0.07 (0.01−0.13)	1.04 (0.96-1.14)	0.99 (0.94-1.04)	1.04 (0.99-1.08)	1.18 (1.13-1.24)	1.03 (0.98-1.07)
*P*_trend_			0.029	0.328	0.550	0.105	<0.001	0.235
Spicy food								
Never	39,456 (45.2)	48.8 (4.2)	0.00	1.00	1.00	1.00	1.00	1.00
<1 d/wk	20,095 (23.0)	48.7 (4.2)	−0.06 (−0.13 to 0.02)	0.90 (0.81-1.00)	1.02 (0.96-1.08)	1.04 (0.99-1.09)	0.94 (0.89-0.99)	0.98 (0.93-1.04)
1-2 d/wk	3,802 (4.3)	48.7 (4.2)	−0.05 (−0.19 to 0.09)	0.84 (0.68-1.04)	1.02 (0.91-1.15)	0.96 (0.88-1.06)	0.87 (0.78-0.97)	0.96 (0.87-1.06)
3-5 d/wk	3,390 (3.9)	48.6 (4.3)	−0.16 (−0.31 to -0.01)	0.98 (0.79-1.21)	0.99 (0.87-1.13)	1.05 (0.95-1.16)	0.87 (0.78-0.97)	0.93 (0.84-1.04)
6-7 d/wk	20,606 (23.6)	48.3 (4.4)	−0.20 (−0.28 to −0.12)	0.92 (0.82-1.02)	1.11 (1.05-1.18)	0.93 (0.88-0.98)	0.78 (0.74-0.83)	0.96 (0.90-1.01)
*P*_trend_			<0.001	0.037	0.001	0.002	<0.001	0.017

ß and OR were adjusted for age, area, education, annual household income, smoking, body mass index (BMI), age at menarche, and number of live births, except for the same variable. When calculating the OR, menopausal age of 48 to 50 years was used as the reference group. *P* < 0.001 did not change after Bonferroni corrections.

CI, confidence interval; OR, odds ratio.

a Adjusted for age at baseline (continuous), except for the age variable.

When comparing the ANM reference category, lower odds of both PM and EM were found in women with higher household income (vs <10k yuan, with ORs range between 0.72 and 0.81, *P*_trend_ < 0.001 for PM and 0.72-0.90, *P*_trend_ < 0.001 for EM); or experienced more induced abortions (vs none): 0.77-0.86 (*P*_trend_ < 0.001) and 0.88-0.94 (*P*_trend_ < 0.001). Low odds for only PM were observed in women with higher education levels (vs no formal school, 0.61-0.74, *P*_trend_ < 0.001), more live births (vs 1, 0.65-0.78, *P*_*trend*_ = 0.002), and later ages at first birth (vs <20 years, 0.68-0.86, *P*_trend_ < 0.001). Other factors such as urban residence, OC ever use, breastfeeding duration, tea drinking, intakes of meat, poultry, seafood, fresh eggs, soybean products, fresh fruits, and dairy products and higher BMI gain per year from age 25 years were also inversely, to some extent, associated with PM and/or EM (Tables 2-6, Fig. 1B–C, Fig. 2B-D).

**TABLE 6:** Adjusted mean age at natural menopause and associations with physical measurement characteristics of women

Characteristics	No. of women (%)	Age at menopause (y), mean (SD)^*a*^	Mean difference ß (95% CI)	<40 yOR (95% CI)	40-44 y OR (95% CI)	45-47 y OR (95% CI)	51-52 y OR (95%CI)	≥53 years OR (95%CI)
Central adiposity								
No (WC <80 cm)	39,480 (45.2)	48.42 (4.30)	0.00	1.00	1.00	1.00	1.00	1.00
Yes (WC ≥80 cm)	47,869 (54.8)	48.83 (4.24)	0.06 (−0.02 to 0.14)	0.91 (0.82-1.01)	0.99 (0.93-1.06)	0.94 (0.90-1.00)	0.98 (0.93-1.04)	1.00 (0.95-1.06)
*P*_trend_			0.131	0.076	0.795	0.030	0.440	0.949
Body mass index (BMI, kg/m^2^)								
Underweight (<18.5)	5,497 (6.3)	48.0 (4.4)	−0.41 (−0.53 to −0.29)	1.00 (0.86-1.17)	1.11 (1.01-1.21)	0.97 (0.89-1.05)	0.77 (0.70-0.85)	0.77 (0.70-0.85)
Normal weight (18.5-23.9)	40,205 (46.0)	48.5 (4.3)	0.00	1.00	1.00	1.00	1.00	1.00
Overweight (24.0-27.9)	29,513 (33.8)	48.8 (4.2)	0.29 (0.23-0.36)	0.97 (0.89-1.07)	0.99 (0.93-1.04)	1.01 (0.97-1.06)	1.11 (1.06-1.17)	1.24 (1.19-1.30)
Obesity (≥28.0)	12,134 (13.9)	49.1 (4.2)	0.49 (0.40-0.57)	0.88 (0.78-1.01)	0.95 (0.88-1.02)	1.03 (0.97-1.10)	1.20 (1.12-1.28)	1.39 (1.31-1.48)
*P*_trend_			<0.001	0.032	0.004	0.048	<0.001	<0.001
BMI change per year from age 25 years (kg/m^2^)								
Lowest quartile (≤−0.030)	16,063 (25.0)	48.6 (4.28)	0.00	1.00	1.00	1.00	1.00	1.00
Lowest quartile to 0	6,111 (9.5)	48.6 (4.19)	0.01 (−0.11 to 0.13)	0.94 (0.79-1.11)	0.93 (0.83-1.03)	0.92 (0.85-1.00)	1.00 (0.91-1.10)	0.92 (0.84-1.01)
0 to second quartile (0.046)	9,959 (15.5)	48.9 (4.16)	0.20 (0.09-0.31)	0.88 (0.75-1.02)	0.89 (0.81-0.98)	0.94 (0.87-1.01)	1.05 (0.97-1.13)	1.01 (0.93-1.09)
Third quartile (0.046-0.122)	16,096 (25.1)	49.0 (4.10)	0.21 (0.11-0.31)	0.86 (0.74-1.00)	0.87 (0.80-0.95)	0.91 (0.84-0.97)	1.07 (0.99-1.15)	0.99 (0.92-1.06)
Highest quartile (>0.122)	16,002 (24.9)	49.1 (4.17)	0.20 (0.08-0.32)	0.98 (0.82-1.17)	0.94 (0.85-1.05)	0.95 (0.87-1.03)	1.08 (0.99-1.18)	1.06 (0.98-1.15)
*P*_trend_			<0.001	0.011	0.017	0.014	0.059	0.084
Hypertension								
No (SBP/DBP<140/90 mm Hg)	40,636 (46.5)	48.5 (4.2)	0.00	1.00	1.00	1.00	1.00	1.00
Yes (SBP/DBP ≥140/90 mm Hg or prior diagnosis)	46,713 (53.5)	48.8 (4.3)	0.17 (0.11-0.23)	1.03 (0.95-1.12)	0.95 (0.91-1.00)	0.96 (0.92-0.99)	1.06 (1.01-1.10)	1.09 (1.04-1.13)
*P*_trend_			<0.001	0.505	0.041	0.022	0.013	<0.001

ß and OR were adjusted for age, area, education, annual household income, smoking, BMI, age at menarche, and number of live births, except for the same variable. When calculating the OR, menopausal age of 48 to 50 years was used as the reference group. *P* < 0.001 did not change after Bonferroni corrections.

BMI, body mass index; CI, confidence interval; DBP, diastolic blood pressure; OR, odds ratio; SBP, systolic blood pressure; WC, waist circumstance.

a Adjusted for age at baseline (continuous), except for the age variable.

Regarding factors associated with LM, compared with the reference category of ANM, higher odds were found in women who had higher BMI (overweight/obesity vs normal weight, OR between 1.24 and 1.39), who were parous, had more pregnancies, occasionally drank alcohol (vs never), more active, intake of vitamin supplement, or had hypertension (Tables 3-6, Fig. 1D).

The sensitivity analyses showed that the ORs for PM, EM, and LM were largely unchanged when the ANM of 48 to 52 years was taken as the reference group.

## DISCUSSION

To our knowledge, this is the first large epidemiological study to comprehensively and simultaneously identify factors related to ANM in China. Based on nearly 90,000 postmenopausal women from ten diverse regions in China, we found various sociodemographic, lifestyle, dietary, and reproductive factors were associated with women's ANM.

Recent evidence has indicated that there is an upward secular trend of ANM in the past decades, with the older generation more likely to experience earlier menopause.^17,18^ Similarly, the present study showed that older women were at higher odds of both PM and EM than their younger counterparts. The mechanism underlying the generation effect on ANM remains unclear, but may be largely due to the economic growth and health status improvement.^18^ Menopausal age has been shown to vary by socioeconomic status (SES) across studies. A recent meta-analysis of 46 studies from 24 countries found that higher education and occupation levels were associated with later ANM.^19^ Similarly, in a national, cross-sectional study of 31,508 Korean women, the authors reported that rural residence, as well as lower household income and education levels were associated with increased risk of PM and/or EM.^20^ The direction of our results supported these studies with findings that women characterized by urban residence, higher education, and household income levels were less likely to experience PM and/or EM. A study of 4,056 women aged 60 to 79 years selected from Latin America and Caribbean showed that manual occupation/being a housewife were associated with earlier menopause.^21^ In our study, housewives consistently showed higher odds of both PM and EM. The relationship of unmarried status with earlier ANM is a relatively consistent observation in the literature. In line with a previous report from Lay et al,^22^ this study also observed that widowed women had a higher odds of EM than those currently married.

To date, the relationship between age at menarche and ANM remains unclear.^23,24^ In line with findings from several large population studies,^25,26^ the present study observed that women with earlier menarche were more likely to have earlier menopause, which may be explained by the fixed follicle pool and therefore possibly fixed number of ovulatory cycles. This mechanism may also explain the relationship between LM and OC use, being parous, increased number of pregnancies and live births, and longer breastfeeding duration, which all disrupt the ovulation cycle to some extent.^27,28^ It was suggested that later age at first birth may be associated with earlier ANM, due to a decline in follicle count and/or sex hormone levels.^29^ In contrast, the present study found that later age at first birth was positively associated with LM, which was also reported from a study in India,^30^ and the possible reasons are unknown. The influence of abortion on menopausal age has been rarely studied with conflicting findings presented. Although no relationship was reported in an Iranian study,^31^ the positive associations of spontaneous and induced abortion with EM and PM odds were reported in a Korean study (137 PM and 281 EM women).^28^ The present study also found that women who reported having three or more spontaneous abortions were at higher odds of EM. Potential explanation was that more spontaneous abortions may accelerate the rate of follicle loss in the decade preceding menopause.^32^ Interestingly, we found that the ANM tended to be later with increasing number of induced abortions, which may be due to the birth cohort effect (ie, induced abortion occurred more often in the younger generations following the One-Child Policy that was introduced in the 1970s).

Prospective evidence has confirmed the role of current regular smoking in accelerating menopause,^33^ consistent with observations in our study. Few studies have explored the potential effect of passive smoking on ANM and the results are inconsistent.^34,35^ The present study found that passive smoking exposure, similarly to active smoking, was also related to earlier menopause. The most important explanation for the accelerating effect of smoking on menopause was that the yielded polycyclic aromatic hydrocarbons would increase the rate of oocyte apoptosis.^36^ Only the Shanghai Women's Health Study of 33,054 Chinese postmenopausal women has investigated the link between tea consumption and menopausal age,^29^ and unlike the null relation reported in their study, our study showed that tea drinking was inversely associated with PM, which was supported by the previously published biological evidence on the antioxidant effects of tea and nonsteroidal estrogenic effects of tea flavonoids.^37,38^ The slightly higher odds of LM in occasional alcohol drinkers that we found is also reported by studies in other countries,^39^ which may be partly due to the increased estrogen levels from alcohol consumption noted in premenopausal women.^40^ Prior evidence for the relationship of physical activity with ANM has been generally mixed. Some studies reported increased physical activity was associated with older age at menopause,^29^ whereas others showed null or inverse associations.^18,41^ In this study, we consistently found that higher physical activity was positively associated with LM. Increased physical activity may delay menopause by causing irregular menstrual cycles, a potential factor associated with later ANM.^42^

The present study found that women with higher consumption of meat, poultry, seafood, eggs, dairy and soybean products had lower odds of having PM and/or EM, which to some extent, confirms the possible delaying effect of protein intake on ANM.^29^ Specifically, the consistently observed positive association between meat and ANM in our cohort and previous studies supported the hypothesis that meat protein may increase episodic releases of luteinizing hormone, follicle stimulating hormone and the length of the menstrual cycle.^43^ In addition, concurring with prior evidence,^44^ the relationship of higher dairy product intake with later ANM observed here may also support the association of dairy product intake with reduction of decline of anti-Mullerian hormone (AMH) level,^45^ a direct marker of ovarian reserve and menopausal age. A similar mechanism may also play a role in our finding that women with higher intake of fresh fruits were less likely to have PM and EM.^45^ In addition, fruits are rich in antioxidants and thought to ameliorate oxidative stress on ovarian follicles and thus affect menopausal age.^46^ The lower odds of EM associated with soybean product intake found in the present study differed from previous studies. An association of soy product intake with earlier menopause was found in a cross-sectional Japanese study^47^ but not in the subsequent follow-up study.^48^ In the Nurses’ Health Study II cohort study of 85,682 premenopausal US women, neither soy nor tofu intake was related to EM over twenty years of follow-up.^49^ The present study also indicated a positive relationship of vitamin intake with LM and women with daily spicy food intake were more likely to have EM. Further studies on observational associations and potential mechanisms are warranted.

In line with prior evidence,^50^ the present study showed that compared to women with normal weight, overweight and obese women tended to have LM. A possible explanation for these findings is that women with higher BMI are expected to have more estrogen supplied by adipose tissue in the later reproductive years.^51^ Many studies have also investigated the associations between dynamic changes of weight or BMI over time and menopausal age, however findings were inconsistent.^52,53^ In this study, we found that BMI gain since young adulthood was inversely associated with EM. In contrast to previous studies showing that premenopausal hypertension was associated with earlier ANM,^53,54^ interestingly, we found that women with clinically identified and screen-detected hypertension were more likely to have LM, which did not materially changed when further confined to premenopausal clinically identified hypertension in women.

The present study has several strengths. This is the first study to comprehensively examine the related factors of ANM in China with the largest sample of postmenopausal women from ten diverse geographic regions in China. In addition, the high quality and completeness of data collection, and the wide adjustment for co-variables simultaneously limit the possible confounding bias in the analyses. However, some limitations exist. The greatest weakness of the study is that the mean age at baseline and menopause among women was 64.5 and 48.7 years, respectively, which means that some baseline exposure factors used for analysis were collected on average 15 years after menopause. Under this circumstance, some of the identified associations may be the result of EM or PM, rather than the cause. For example, a woman who started smoking at age 60 would be considered a smoker for this analysis and contribute to findings that smoking is associated with an earlier ANM, even if she started smoking many years after menopause. Thus, these factors we examined and their associations with ANM need to be interpreted with great caution and identified in longitudinal studies. In addition, the data of menopause and most exposure variables collected relied on subjective self-reports thus recall bias may exist. Although evidence has shown that recalled and actual menopausal age is reasonably well correlated,^55^ considering an average of 15 years have passed since menopause, the recall bias of ANM is inevitable. Furthermore, generally high or moderate agreements on the self-reported exposure factors between the baseline survey and resurvey suggested recall bias may be relatively small in CKB. Taking age at menarche that was recalled decades later as an example, the intraclass correlation coefficient was 0.84 between the baseline survey and the resurvey.^56^

## CONCLUSIONS

In summary, this study indicates that a wide range of sociodemographic, lifestyle, dietary, and reproductive factors are found to be related to PM, EM, and LM in Chinese women. As the ANM has implications for several health outcomes, the findings in this study also provide support for early monitoring of women who are at high risk for chronic diseases occurring in later life.

## Supplementary Material

Supplemental Digital Content
