# Supplementary material for: Factors related to age at natural menopause in China: results from the China Kadoorie Biobank
Source: Menopause. 2021 Aug 2;28(10):1130–42. doi: 10.1097/GME.0000000000001829 (PMC8462451; doi:10.1097/GME.0000000000001829)
Supplement: Supplemental Digital Content [file menop-28-1130-s001.pdf]

June 10, 2004

Kadoorie Study of Chronic Disease in China

【Questionnaire】

Clinical Trial Service Unit & Epidemiological Studies Unit,
Nuffield Department of Clinical Medicine, University of Oxford, UK
&
Chinese Centre for Disease Control and Prevention, Beijing, P. R. China

Section 1: Background information

1.1 Study ID: ☐☐☐☐☐☐☐☐☐☐ *(The date & time of interview will be recorded by computer)*

1.2 Name____________**, Sex** : Male ☐ Female ☐ , **Name of spouse**________________

1.3 Date of birth: ☐☐☐☐ Year ☐☐ Month ☐☐ Day

1.4 National ID number (if no, put #) ☐☐☐☐☐☐☐☐☐☐☐☐☐☐☐☐☐☐

1.5 Home address : _____ Province _____ City_________ District/County _____ Street/Village

Home telephone: Not available ☐ , Yes: ☐☐☐☐ – ☐☐☐☐☐☐☐☐☐☐☐

1.6 What is the highest level of school education you ever received?

- ☐ No formal school
- ☐ Primary School
- ☐ Middle School
- ☐ High School
- ☐ Technical school / college
- ☐ University

1.7 What is your current occupation?

- ☐ Agriculture & related workers
- ☐ Factory worker
- ☐ Administrator / manager
- ☐ Professional / technical
- ☐ Sales & service workers
- ☐ Retired
- ☐ House wife / husband
- ☐ Self-employed
- ☐ Unemployed
- ☐ Other or not stated

1.8 How many people living together in the household? ☐☐ persons

1.9 What is your current marital status?

- ☐ Married
- ☐ Widowed
- ☐ Separated / divorced
- ☐ Never married

1.10 What is the total income last year in your household?

- ☐ <2,500 yuan
- ☐ 2,500-4,999 yuan
- ☐ 5,000-9,999 yuan
- ☐ 10,000-19,999 yuan
- ☐ 20,000-34,999 yuan
- ☐ ≥35,000 yuan

1.11 Do you have any health care cover and following items in your household?

Yes	No	
☐	☐	Health care cover
☐	☐	Own house / apartment
☐	☐	Toilet for private use
☐	☐	Telephone or mobile phone
☐	☐	Motor vehicle (e.g. car or motorbike)
☐	☐	Holiday during last five years

Section 2: Tea drinking

2.1 During the past 12 months, how often did you drink any tea?

- ☐ Never or almost never
- ☐ Only occasionally
- ☐ Only at certain seasons
- ☐ Every month but less than weekly
- ☐ Usually at least once a week → ***Go to Q2.3*** (were2.2)

2.2(were 2.1a) **In the past, did you ever have a period of at least 1 year during which you usually drank tea at least once a week?**

- ☐ Yes, → (were 2.1b) if so, how long ago did it end? [|] Years } ***Go to section 3***
- ☐ No

2.3(were 2.2) **During the past 12 months, on how many days did you drink tea in a typical week?**

- ☐ 1-2 days/week
- ☐ 3-5 days/week
- ☐ Daily or almost every day

2.4(were2.3)**At about what age did you start drinking tea in most weeks?** ……… [|] Years

---

2.5(were2.4)**On days when you drink tea, how many cups do you usually drink?** (choose one only)

Green /Jasmine tea ……………………………………………… [|] cups/day

Oolong tea ……………………………………………… [|] cups/day

Black tea ……………………………………………… [|] cups/day

Other tea ……………………………………………… [|] cups/day

2.6(were2.5) **How often do you change tea leaves during a day?** ………… [|] times

---

2.7(were2.6) **About how much tea leaves do you usually add each time?** [|] Grams

---

2.8(were2.7) **What strength of tea do you usually prefer to drink?**

- ☐ Weak
- ☐ Moderate
- ☐ Strong

2.9(were2.8) **At about what temperature do you usually drink your tea?**

- ☐ Room temperature / warm
- ☐ Hot
- ☐ Burning hot

2.10(were2.9)**Has your tea consumption changed significantly compared with that some years ago?** ☐ About the same as before, ☐ Has increased a lot, ☐ Has decreased a lot

---

Section 3: Alcohol consumption

3.1(were3.0) **Have you drunk any alcohol today?** ☐ Yes , ☐ No

3.2(were3.1)**During the past 12 months, how often did you drink any alcohol?**

- ☐ Never or almost never
- ☐ Only occasionally
- ☐ Only at certain seasons
- ☐ Every month but less than weekly
- ☐ Usually at least once a week → *Go to Q3.4* (wereQ3.2)

3.3(were3.1a)**In the past, did you ever have a period of at least 1 year, during which you usually drank some alcohol at least once a week?**

- ☐ Yes, →(were 3.1b) If so, how long ago did it e[illegible] ☐☐ Years
- ☐ No

} *Go to section 4*

3.4(were3.2)**During the past 12 months, on how many days did you drink alcohol in a typical week?**

- ☐ 1-2 days/week
- ☐ 3-5 days/week
- ☐ Daily or almost every day

3.5(were3.3)**At about what age did you start drinking some alcohol in most weeks?** ☐☐ Years

---

3.6(were3.4) **On days when you drink, how much alcohol do you usually drink in a day?**
(Can choose up to 3 types of alcohol for special occasions; for beer, 1 large bottle=2 small ones**)**

Alcohol type	On a typical day (choose one)	On a special day when you drink a lot	Last time when you drank
Beer (large)	☐☐ Bottle	☐☐ Bottle	☐☐ Bottle
Rice Wine	☐☐ liang	☐☐ liang	☐☐ liang
Wine	☐☐ liang	☐☐ liang	☐☐ liang
Spirit (≥50% alcohol)	☐☐ liang	☐☐ liang	☐☐ liang
Spirit (<50% alcohol)	☐☐ liang	☐☐ liang	☐☐ liang

3.7(were3.5)**On a typical day when you drink alcohol, when do you usually take the drink?**

- ☐ Usually drink with the meal
- ☐ Usually drink between or after the meals
- ☐ No regular pattern

3.8(were3.6)**After drinking alcohol, do you usually experience hot flushes or dizziness?**

- ☐ Yes, soon after first mouthful
- ☐ Yes, after drinking small amount of alcohol
- ☐ Yes, but only after drinking large amount of alcohol
- ☐ No

3.9(were3.7)**During the past month, how often have you drunk alcohol in the morning?**

- ☐ Never
- ☐ <1 day/week
- ☐ A few days a week
- ☐ Daily or almost daily

3.10(were3.8)**During the past month, have you ever had the following experiences?**

Yes	No	
☐	☐	Unable to work or to do anything because of drinking
☐	☐	Felt depressed, irritated or couldn't control yourself after drinking
☐	☐	Could not keep away from drinking
☐	☐	Had shakes when you stopped drinking

3.11(were3.9) **Has your alcohol consumption changed significantly compared with that some years ago?**

- ☐ About the same as before
- ☐ Has increased a lot
- ☐ Has decreased a lot

Section 4: Smoking history

4.1(were4.0) **Have you smoked any tobacco today?**☐ **Yes ,** ☐ **No,** →if yes, how many: __ total, __in last hour

4.2(were4.1)**How often do you smoke tobacco now?**

- ☐ Do not smoke now
- ☐ Only occasionally
- ☐ Yes, on most days }
- ☐ Yes, daily or almost every day } → ***Go to Q4.7*** (were Q4.5)

4.3(were4.2)**In the past, how frequently did you smoke?**

- ☐ Did not smoke
- ☐ Smoked only occasionally
- ☐ Smoked on most days }
- ☐ Smoked daily or almost every day } → ***Go to Q 4.5*** (were Q4.3)

4.4(were4.2a)**In your life time, have you smoked a total of at least 100 cigarettes or equivalent?**

- ☐ Yes }
- ☐ No } ➔ ***Please go to section 5***

4.5(were4.3)**How many years ago did you last stop smoking regularly?** ☐☐ Years

4.6(were4.4)**What was your main reason for stopping?**

- ☐ Physical illness that you already had
- ☐ Money
- ☐ Health concerns (about future illness)
- ☐ Family against
- ☐ Other

4.7(were4.5)**At about what age did you first start smoking on most days?** ☐☐ Years

4.8(were4.6)**What tobacco did you use when you first started smoking on most days?**

Mainly cigarette ☐, Mainly non-cigarette ☐, Mixed types ☐

↳ (4.6a) If so, have you always smoked some cigarettes on most days, never having a month or more without them? Yes ☐, No ☐

4.9(were4.7)**How much tobacco do you usually smoke (or did you smoke before giving up)?**

Filter cigarettes (factory)	☐☐	number/day
Non-filter cigarettes (factory)..	☐☐	number/day
Hand-rolled cigarettes	☐☐	liang/month
Pipe or water pipe	☐☐	liang/month
Cigars	☐☐	number/day

4.10(were4.9) **How deeply do you usually inhale the smoke?**

- ☐ Mouth only
- ☐ Throat
- ☐ Lung ➔ 4.9a Have you always inhaled the smoke into your lung when smoking? Yes ☐, No ☐

4.11(were4.10)**Has your tobacco consumption changed significantly compared with that some years ago?**

☐ About the same as before, ☐ Has increased a lot, ☐ Has decreased a lot

Section 5: Diet

5.1(were5.2)**During the past 12 months, about how often did you eat the following foods?**

	Daily	4-6 days per week	1-3 days per week	Monthly	Never/rarely
Rice	☐	☐	☐	☐	☐
Wheat	☐	☐	☐	☐	☐
Other staple food (corn, millet etc.)	☐	☐	☐	☐	☐
Meat	☐	☐	☐	☐	☐
Poultry	☐	☐	☐	☐	☐
Fish/sea food	☐	☐	☐	☐	☐
Fresh eggs	☐	☐	☐	☐	☐
Fresh vegetables	☐	☐	☐	☐	☐
Soybean products	☐	☐	☐	☐	☐
Preserved vegetables	☐	☐	☐	☐	☐
Fresh fruit	☐	☐	☐	☐	☐
Dairy products (milk, yogurt)	☐	☐	☐	☐	☐

5.2(were5.3)**During the past 12 months, have you taken the following supplements regularly?**

Yes	No	
☐	☐	Fish oil/cod liver oil
☐	☐	Vitamins
☐	☐	Calcium/iron/zinc
☐	☐	Ginshen (at least 5 or more times during a year)
☐	☐	Other herbal products

5.3(were5.3a)**Have you ever experienced any severe food shortage?** ☐Yes, ☐ No → ***Go to Q5.6(wereQ5.3c)***

5.4(were5.3a1)**What year was the worst food shortage you experienced?** ______________ **years**

5.5(were5.3b)**During the most severe food shortage you experienced:**

5.5.1− did you lose weight? ☐ Yes, ☐ No, → If yes, about how much? ______ *jin,*

5.5.2− did you develop any specific disease related to food shortage? ☐ Yes, ☐ No

5.6(were5.3c)**How many years have you had a refrigerator in your home?** ☐☐ Years

---

5.7(were5.4) **During the past month, about how often did you eat hot spicy food?**

☐ Never or almost never
☐ Only occasionally
} → ***Go to section 6***
☐ 1-2 days/week
☐ 3-5 days/week
☐ Daily or almost every day

5.8(were5.5)**At what age did you start to eat spicy food at least once a week?** ☐☐ Years

5.9(were5.6)**What strength of spicy food do you usually prefer to eat?**

☐ Weak, ☐ Moderate, ☐ Strong

5.10(were5.7)**On day when you eat spicy food, what are the main sources of spice usually used?**

Yes	No	
☐	☐	Chili sauce
☐	☐	Chili oil
☐	☐	Dried chili pepper
☐	☐	Fresh chili pepper
☐	☐	Other or don’t know

Section 6: Passive smoking & indoor air pollution

6.1 Have you ever lived with smoker in the same house for at least 6 months?

- ☐ Never
- ☐ Yes, but not now
- ☐ Yes, at present

} → ***If yes, duration of living together*** [ ][ ] years

6.2 How frequently are you exposed to other people's tobacco smoke either at home, workplace or in public places? *(i.e. a minimum of 5 consecutive minutes each time)*

- ☐ Never or almost never
- ☐ Occasionally (<1 time/week)

} → ***Go to Q6.4***

- ☐ 1-2 days/week
- ☐ 3-5 days/week
- ☐ Daily or almost every day

6.3 What is the usual duration of your exposure per week? [ ][ ] Hours

---

6.4 During past year, how long did you store pesticides at home? [ ][ ] Months

---

6.5(were6.4a)**Please tell us the duration you lived in 3 most recent houses** (each for at least 1 year)?

Present house [ ][ ] years

Previous house [ ][ ] years

The house before previous [ ][ ] years

6.6(were6.5) **In your present & two previous houses, how often did you cook at home?**

- ☐ Daily
- ☐ Weekly
- ☐ Monthly
- ☐ Never/Rarely → ***Go to Q6.10*** **(wereQ6.8)**
- ☐ No cooking facility → ***Go to Q6.11*** (wereQ6.11)

6.7(were6.6)**In your present & two previous houses, what was the main cooking fuel used?**

- ☐ Gas
- ☐ Coal
- ☐ Wood
- ☐ Eletricity
- ☐ Other

6.8(were6.7)**In your present & two previous houses, what was the main cooking oil used?**

- ☐ Rapeseed
- ☐ Peanut
- ☐ Soybean
- ☐ Lard
- ☐ Other

6.9(were6.7a)**How much time have you spent on cooking so far today?** [ ][ ] minutes

---

6.10(were6.8)**In your present & two previous houses, did your stove(s) all have a chimney / extractor?**

☐ Yes ☐ Not all stoves ☐ No

6.11(were6.9)**In your present & two previous houses, was your stove always kept under slow burning throughout the day?**

☐ Yes, always ☐ Yes, sometimes ☐ No → if ticked, ***Go to Q6.14***

6.12(were 6.9a) **If yes , types of the fuel most commonly used?**

☐ Smokeless coal ☐ Coal brick / Coalite

☐ Smoky coal ☐ Other

6.13(were 6.9b) **And , the place where stove was usually kept?**

☐ Inside the house ☐ Outside the house

6.14(were6.11)**In winter, did you normally heat your house?**

☐ Yes, ☐ No

6.15 If yes, what was the main heating fuel used?

☐ Central heating ☐ Wood

☐ Gas ☐ Electricity

☐ Coal ☐ Other

6.16(were6.12)**From what year did the inside of your house tend to be coal-smoky in winter?**

☐ Never → if ticked, ***Go to section7***

☐ Ever since childhood

☐ Since the year: _________ year

6.17(were6.13)**In what year did the inside of your house stop being really coal-smoky in winter?**

☐ In the year: _________ year

☐ Still is

Section 7: Personal & family medical history

7.1 How is your current general health status?

7.1.1 Self-rated health status?

☐ Excellent
☐ Good
☐ Fair
☐ Poor

7.1.2. Compared to someone of your own age?

☐ Better
☐ About the same
☐ Worse
☐ Don't know

7.2 If you were walking on level ground with other healthy people of the same age, would you usually:

7.2.1 Become short of breath? ☐ Yes ☐ No ☐ Disabled

7.2.2 Slow down due to chest discomfort? ☐ Yes ☐ No ☐ Disabled

7.3 During the past 12 months, have you usually had the following symptoms?

7.3.1 Cough frequently?

☐ No
☐ Yes, for <3 months
☐ Yes, for ≥3months

7.3.2 Cough up sputum after getting up in the morning?

☐☐ No
☐☐ Yes, for <3 months
☐☐ Yes, for ≥3 months

7.4 Has a doctor EVER told you that you had had the following disease?

	Diagnosed disease?		Age at first diagnosis	Still on treatment?	
	Yes	No		Yes	No
Diabetes	☐	☐	______	☐	☐
CHD	☐	☐	______	☐	☐
Stroke or TIA	☐	☐	______	☐	☐
Hypertension	☐	☐	______	☐	☐
Rheumatic heart dis.	☐	☐	______	☐	☐
TB	☐	☐	______	☐	☐
Emphysema/bronchitis	☐	☐	______	☐	☐
Asthma	☐	☐	______	☐	☐
Cirrhosis/chronic hepatitis	☐	☐	______	☐	☐
Peptic ulcer	☐	☐	______	☐	☐
Gallstone/gallbladder dis.	☐	☐	______	☐	☐
Kidney disease	☐	☐	______	☐	☐
Fracture	☐	☐	______	☐	☐
Rheumatoid arthritis	☐	☐	______	☐	☐
Psychiatric disorders	☐	☐	______	☐	☐
Neurasthenia	☐	☐	______	☐	☐
Head injury	☐	☐	______	☐	☐
Cancer*	☐	☐	______	☐	☐

For CHD, stroke, and hypertension what is the current medication:

1. Aspirin
2. ACE-I
3. Beta-blocker
4. Statins
5. Diuretics
6. Ca^{++} antagonist

& for diabetes, the above list plus

7. Chlorpropamide or metformin
8. Insulin

***If yes, please indicate the site of cancer** ☐ *(If more than one, choose the one that occurred first)*

0. Lung 1. Esophagus 2. Stomach 3. Liver 4. Intestine 5. Breast 6. Prostate 7. Cervix 8. Other

7.5 **Have many blood transfusions have you ever received?** *(If none, put 0)* ☐☐ times

7.6(were7.5a)**How many times have you ever donated blood for financial payment?**

(If none, put 0) ☐☐ times

7.7(were7.6)**About how often do you have bowel movements each week?**

- ☐ More than once on most days
- ☐ About daily
- ☐ Once every 2-3 days
- ☐ Less than 3 times a week

7.8(were7.7)**How often do your gums bleed when you brush your teeth?**

- ☐ Occasionally, rarely or never
- ☐ Sometimes
- ☐ Always
- ☐ Brush teeth rarely or never

7.9(were7.8)**How many brothers & sisters do you have?***(Including half siblings. If unknown, put#)* ☐☐

7.10(were7.9)**How many children do you have?** *(Including only biological ones)* ☐☐

7.11(were7.9a)**Is your mother still alive?**

- ☐ Yes → If ticked, current age: ☐☐
- ☐ No → If ticked, age at death: ☐☐
- ☐ Unknown

7.12(were7.9b)**Is your father still alive?**

- ☐ Yes → If ticked, current age: ☐☐
- ☐ No → If ticked, age at death: ☐☐
- ☐ Unknown

7.13(were7.10) **Did any of your parents, siblings or children have following diseases?** *(For sibling and children, please record the number with disease)*

	**Stroke**	**Heart attack**	**Diabetes**	**Mental disorder**	**Cancer**
**Mother** *(tick box)*	☐	☐	☐	☐	☐
**Father** *(tick box)*	☐	☐	☐	☐	☐
**Siblings** *(inclu. half)*	☐	☐	☐	☐	☐
**Children**	☐	☐	☐	☐	☐

Section 8U: Physical activities (Urban)

8.1 During the past 12 months, how active were you at work?

- ☐ Mainly sedentary (e.g. office worker)
- ☐ Standing occupation (e.g. guard, shop assistant)
- ☐ Manual work (e.g. plumber, carpenter)
- ☐ Heavy manual work (e.g. miner, construction worker)
- ☐ Retired or housewife/husband or unemployed or disabled → ***If ticked, please go to Q8.8***

8.2(were8.1a) **In a typical week, about how many hours did you usually work? _______ hours**

8.3(were8.2) **During the past 12 months, how did you usually get to work?**

- ☐ Mainly walk
- ☐ By motorbike
- ☐ By bicycle
- ☐ By bus/car/ferry/train
- ☐ Mainly stay at home or work near home
  ↳***If ticked, please go to Q8.8***

8.4(were8.3) **How much time did you spend each day on journey to & from work? _____minutes**

Section 8F: Physical activities (New section for rural farmers)

8.1 During the past 12 months, did your farming work change seasonally?

- ☐ No → ***go to Q8.3***
- ☐ Yes

8.2 During the farming season in the last 12 months:

8.2.1 How many months did it usually last? ☐☐ month

8.2.2 What types of work did it usually involve?

☐ manual ☐ Semi-mechanized ☐ Fully mechanized

8.2.3 How many hours did you usually work each day? ☐☐ hours

8.2.4 Of which , how many hours did you sweat or have a much faster heartbeat?

☐☐ hours

8.3 In a typical week, how many hours did you usually work in the field? ☐☐ hours

---

8.4 Apart from agriculture work, did you have any other job?

- ☐ No → ***go to Q8.7***
- ☐ Yes

8.5 How active were you at work with other job?

- ☐ Mainly sedentary
- ☐ Mainly standing
- ☐ Mainly general manual work
- ☐ Mainly heavy manual work

8.6 In a typical week, about how many hours did you work at other job? ☐☐ hours

---

8.7 In a typical day how much time did you usually spend on the journey to and from work on foot or by bicycle? ☐☐☐ minutes

---

Section 8C: Physical activities (Common to both rural farmers and urban)

8.8 During the past 12 months, how often did you do exercise in your leisure time?

☐ Never or almost never } → ***If ticked, please go to Q8.11***
☐ 1-3 times/month
☐ 3-5 times/week
☐ 1-2 times/week
☐ Daily or almost every day

8.9 What is your main type of exercise? *(tick one box only)*

☐ Taichi / Qigong
☐ Walking
☐ Jogging/aerobic exercise
☐ Swimming
☐ Ball games (basketball, table tennis, etc)
☐ Other (eg. mountain climbing)

8.10 About how many hours per week did you do such exercise in leisure time? ___ hours

---

8.11 In a typical week during the past 12 months, how often did you sweat or have a much faster heartbeat because of heavy physical activities/exercise?

☐ Never or almost never } → ***If ticked, please go to Q8.13***
☐ <1 time / week
☐ 3-5 times/week
☐ 1-2 times/week
☐ Daily or almost every day

8.12 About how many hours per week did you do such activities? _______ hours

---

8.13 About how many hours per week did you do house work? _______ hours

8.14 About how many hours per week did you watch TV or read? _______hours

---

8.15 During the past 12 months, has your weight changed significantly?

☐ About the same as before ☐ Yes, gained ≥2.5 kg ☐ Yes, lost ≥2.5 kg

---

8.16 Have you tried to reduce weight in the past 12 months? No ☐, Yes ☐

8.17 How much did you weigh when you were at age 25? (If unknown put #) |__|__|__| jin

Section 9: Reproductive history (for women)

9.1 How old were you when you had your first menstrual period? ☐☐ Year

9.2 Have you had your menopause?

- ☐ No
- ☐ Yes, currently
- ☐ Yes, had menopause → If so, age of completion of menopause: ☐☐ Year

9.3 How many times have you ever been pregnant? (if none, put 0. Go to Q9.6) ☐☐ times

— Of which,

Live birth ☐☐ times → If none, Go to Q9.5 (wereQ9.6)

Still birth ☐☐ times, Spontaneous abortion ☐☐ times, Induced abortion ☐☐ times

9.4 Age and length of breastfeeding at each live birth (twins=one birth)?

Live Birth	Age at end of pregnancy	Months of breastfeeding
1st	☐☐	☐☐
2nd	☐☐	☐☐
3rd	☐☐	☐☐
4th	☐☐	☐☐
5th	☐☐	☐☐

9.5(were9.6)**Have you ever used oral contraceptive pills?**

- ☐ Never → ***If ticked, please go to Q9.8*** (were Q9.9)
- ☐ Past use → **if ticked,** age when you last stopped the pill: ☐☐ Year
- ☐ Current use

9.6(were9.7)**How old were you when you first used oral contraceptives**? ☐☐ Year

9.7(were9.8)**For how long altogether have you used oral contraceptives?** ☐☐ Year

9.8(were9.9)**Have you had a hysterectomy**?

☐ No , ☐ Yes → If yes, age when you had the operation ☐☐ Year

9.9(were9.10)**Have you had one or both ovaries removed?**

☐ No , ☐ Yes → If yes, age when you had the most recent operation ☐☐ Year

9.10(were9.11) **Have you ever had surgery to remove a breast lump?**

☐ No, ☐ Yes → If yes, age when you most recently had the operation ☐☐ Year

Section 10: Sleeping, mood & mental situation

10.1 In general, how satisfied are you with your life?

- ☐ Very satisfied
- ☐ Satisfied
- ☐ Neither satisfied nor dissatisfied
- ☐ Unsatisfied
- ☐ Very unsatisfied

10.2 Over the past two years have you had any of the following major events in your life?

Yes	No		Yes	No	
☐	☐	Marital separation/divorce	☐	☐	Major injury or traffic accident
☐	☐	Loss of job/retirement	☐	☐	Death /major illness of spouse
☐	☐	Business bankrupt	☐	☐	Death/major illness of other close family member
☐	☐	Violence	☐	☐	Major natural disaster (e.g. flood & drought)
☐	☐	Major conflict within family	☐	☐	Loss of income / living on debt

---

10.3 During the past month, did you have any of the following for ≥3 days each week?

Yes	No	
☐	☐	Taking >30 minutes to fall asleep after going to bed or waking up in the middle of the night
☐	☐	Waking up early and not being able to go back to sleep
☐	☐	Needing to take medicine (including herbal or sleeping pills) at least once a week to help sleep
☐	☐	Having difficulty staying alert while at work, eating or meeting people during daytime

---

10.4 Do you usually take a daytime nap? ☐ Yes usually, ☐ Yes ,but only in summer , ☐ No

10.5(were10.4a) **Do you snore during sleep?** ☐ Yes, Frequently, ☐ Yes, Sometimes, ☐ No / Don't know

10.6(were10.5)**How many hours do you typically sleep per day (incl. naps)?** ☐☐ Hours

---

10.7(were10.6)**During the past 12 months, have you had following situations for 2 or more weeks?**
(If answer yes to any of the questions, complete CIDI-A)

Yes	No	
☐	☐	Feeling much more sad, or depressed than usual
☐	☐	Loss of interest in most things like hobbies or activities that usually give you pleasure
☐	☐	Felt so hopeless that you had no appetite to eat even your favourite food
☐	☐	Feeling worthless and useless, everything went wrong was your fault and life is very difficult that there was no way out

---

10.8(were10.7) **During the past 12 months, have you experienced the following situations?**

Yes	No	
☐	☐	Having a period lasting <u>one month or longer</u> when <u>most of time</u> you felt worried, tense, or anxious and it interfered your life *(if yes, complete CIDI-B)*
☐	☐	Having a pain or discomfort in your body lasting ≥3 months that interfered with your life
☐	☐	Having had a spell or an attack when all of sudden felt frightened, anxious, or very uneasy
☐	☐	Having had inexplicable strong fear in situations such as closed space (cave, elevator, airplane etc), in the crowds or public such that you would avoid such situations

---

Section 11: Physical examination

11.1	**Standing height** (without shoes)	[ ][ ][ ][.]	m
11.2	**Sitting height**	[ ][ ][ ][.]	cm
11.3	**Waist**	[ ][ ][ ][.]	cm
11.4	**Hip**	[ ][ ][ ][.]	cm
11.5	**Weight** (without shoes, but in light clothing)	[ ][ ][ ][.]	Kg
11.6	**BMI**	[ ][ ][.]	Kg/m^2
11.7	**Impedance**	[ ][ ][ ]	Ω
11.8	**Fat %** (with one decimal point)	[ ][ ][.]	

Staff code [ ][ ][ ]

11.9 Did you take any drugs to lower blood pressure in the last 2 days? ☐ Yes ☐ No

11.10 Blood pressure & heart rate (to be measured after 5 minutes in the seated position)

	First	Second	
**SBP**	[ ][ ][ ]	[ ][ ][ ]	mmHg
**DBP**	[ ][ ][ ]	[ ][ ][ ]	mmHg
**Heart rate**	[ ][ ][ ]	[ ][ ][ ]	bpm

11.11(were11.10a)**Hours since last ate anything (ignore any drinks)? _______ hours**

11.12(were11.11)**Blood sample collected:** Yes (, . Failed (

Staff code [ ][ ][ ]

11.13 (were11. 12) **Lung function & CO levels:**

	First	Second	
**CO**	[ ][ ][ ]	[ ][ ][ ]	
**%COHB**	[ ][ ][.][ ][ ]	[ ][ ][.][ ][ ]	%
**FEV1**	[ ][.][ ][ ]	[ ][.][ ][ ]	Liter
**FVC**	[ ][.][ ][ ]	[ ][.][ ][ ]	Liter

Staff code [ ][ ][ ]

11.14(were11.13)Assessment of subject's cooperation and the reliability of data collected?

Assessment of subject's cooperation?

- ☐ Good
- ☐ Fair
- ☐ Poor

11.15 Assessment of the reliability of the information collected?

- ☐ Good
- ☐ Fair
- ☐ Poor

Date of interview ______Year______Month____Day, **Signature of interviewer__________**
